# Knowledge and sources of accessing sexual and reproductive health information among visually impaired women in Ghana

**DOI:** 10.1186/s13104-019-4568-6

**Published:** 2019-08-20

**Authors:** Eric Badu, Isaac Mensah, Naomi Gyamfi, Elvis Agyei-Okyere, Abodey Eric, Josephine Adusei-Nkrumah

**Affiliations:** 1 Department of Health Promotion and Disability Studies, School of Public Health, Kwame Nkrumah University of Science and Technology, Kumasi, Ghana; 2 Isaac Mensah, Department of Special Education, University of Education, Winneaba, Ghana; 3 Kwame Nkrumah University of Science and Technology, Accra, Ghana; 4 Eric Abodey, Department of Education and Psychology Studies, University of Cape Coast, Cape Coast, Ghana

**Keywords:** Sexual health, Reproductive health, Health information, Women, Ghana

## Abstract

**Objective:**

This study aims to explore the knowledge and sources of accessing sexual and reproductive health services and care information among visually impaired women in Ghana. Qualitative data involving in-depth interviews and focus group discussions were conducted among 21 visually impaired women selected through purposive and snowballing sampling techniques. Thematic Analysis was used to analyse the data.

**Results:**

The study showed that visually impaired women were active seekers of SRH information (knowledgeable about SRH information and understand the relevance of accessing such information) and passive recipient of SRH information (through formal and informal sources). However, some contextual factors (lack of family and caregivers support services) created barriers for visually impaired women when accessing SRH information. Government advocacy and awareness campaigns on SRH services should consider both formal and informal sources. Family caregivers and SRH health centres should provide adequate support services for visually impaired women regarding information on SRH service.

## Introduction

Health information seeking behavior generally outlines the activities that involve the search and use of information on diseases and health related activities [1]. Health information highlights the provision of information about health services and providers, thus making patients aware of the health services available [2]. Indeed, understanding consumer information seeking behavior can provide health information experts with valuable information to improve the health and well-being of a population [1]. Several theoretical models have been conceptualized to facilitate the understanding of health information seeking behaviour in different population [1, 3, 4]. Examples of the theoretical models are the stress, appraisal and coping, trans theoretical model (TTM) of health behavior change, model of health information seeking behaviours, the health information acquisition model, and monitoring and blunting hypothesis [1, 3, 4].

The current paper adapts the Longo’s et al. expanded model of health information seeking behaviours which explains the nature, source and use of health information related to chronic disease [1, 3]. The theory is adapted to explain the knowledge and sources of accessing sexual and reproductive health information among visually impaired women. Specifically, the theory has four dimensions namely; personal factors (eg demographic, socioeconomic, health history, culture, language, attitudes and interpersonal communication motives), contextual factors (eg. healthcare structure, delivery of healthcare, information environment, information seeking for self, family members, interpersonal social supports and networks), active information seeking and passive receipt of information [1, 3]. Active information seeking describes the extent of consumers’ awareness, access and use of health information to make health decision. In addition, passive receipt of information explains the extent at which consumers receives information from traditional formal sources to inform health decision. The increase in active information-seeking and passive receipt of health information could have a significant effect on the SRH outcome of visually impaired women (eg. satisfaction, quality of life, improved psychological and social well-being, better SRH status).

Globally, past evidence suggests that women with disability lag behind as far as accessing SRH service information is concerned. Particularly, women with disability in Low and Middle Income Countries (LMICs) face substantial barriers in accessing information on SRH services [5–10]. These barriers are mostly related to the socio-cultural beliefs, financial difficulties [11, 12], physical and environmental issues [5, 7] as well as ineffective health systems [12, 13].

In Ghana, several efforts have been embarked on to address the needs of persons with disability of which visually impaired women are a part of. The government in 2006 passed the ACT 715, which is Persons with Disability Act to improve the living conditions of people with disability. Also, it ratified the UNCRPD in 2012 to ensure the inclusion of people with disabilities. These efforts by the government present a new era of improving the social, economic and political well-being of people with disabilities. That notwithstanding, women with disability in Ghana continue to face substantial barriers in accessing social services including health [14–17], education [18, 19] and economic activities [20–23]. More specifically, women with disability do not only face barriers in accessing SRH services and care but also difficulty in getting health information.

Recently, several empirical studies have been undertaken on the accessibility of health services for people with disability. These studies focus largely on the barriers to accessing health services for people with physical disabilities [14, 16, 17]. However, little empirical evidence is known about SRH services and care information. The few empirical studies on SRH services and care information is limited to those with hearing impairment [24–27] and issues regarding maternal health [7]. No study has explored the SRH information needs for visually impaired women. This study aims to explore the knowledge and sources of SRH services and care information among visually impaired women in Ghana.

## Main text

### Methodology

The study was conducted in the Ashanti (Kumasi Metropolis, Bekwai Municipality, and the Amansie West District) and Brong Ahafo Regions (Wenchi Municipality) of Ghana. These two regions were purposively selected due to their geographical location (middle belt), which have mixed features of the southern and northern Ghana [15]. This qualitative study was conducted between September 2016 and May 2017 [15]. The qualitative approach involved in-depth interviews and Focus Group Discussion [15]. This method helped to explore the subjective experiences of visually impaired women [15]. The study used purposive sampling and snowballing techniques to select 21 visually impaired women [15]. The research team attended two separate meetings of the Ghana Blind Union (GBU) and Ghana Federation of Disability Organizations (GFD) to recruit participants [15]. During these meetings, all visually impaired women aged 16 years and above were recruited. In the Amansie West District specifically, a snowballing approach was used to zone the communities in order to locate visually impaired women [15, 28, 29].

### Data collection procedure

A total of 21 visually impaired women (16 years and above) were recruited. Out of this total, seven were from the Bekwai Municipality, six from the Kumasi Metropolis, two from the Amansie West District and six from Wenchi Municipality. Nine IDIs (9 participants) were conducted with visually impaired women and two FGDs (six participants each) [15]. The data were collected until a saturation point was reached [30]. A structured interview guide was used to collect the in-depth interviews and FGDs. The interview guide was developed based on variables from previous theories and literature reviews [5–10]. The interviews were conducted by two research assistants, where one facilitated the interviews and the other monitored and took notes of information that could not be recorded [15]. The research team briefed participants about the research procedures, objectives and consent [15]. Permission was sought for interviews to be recorded. The interview guide was developed in English, however, the interviews were held in the local dialect (Twi) [15]. Each interview took approximately 45 – 60 min.

### Data analysis

The study used thematic analysis to analyse the data. The thematic analysis involved a six-staged process which included familiarizing, generating initial codes, searching for a theme, reviewing themes, defining and naming themes and producing the report [29, 31–33]. All segments of the audio-recorded interviews were transcribed verbatim into a word document. The research team read through the transcribed data several times to generate initial codes and themes (see Table).

### Results

#### Background information

The background information of participants is presented in Table 1. The median and mean ages of the participants were 50 and 42 years respectively [15].

**Table 1:** Background information of participants

Variable	Frequency	Percentage
Age^a^		
16–39	9	42.86
≥ 40 years	12	57.14
Marital status		
Single	9	45.00
Married	5	25.00
Separated	2	10.00
Widow	4	20.00
Education		
No education	6	28.57
JHS	4	19.05
SHS	8	38.10
Tertiary	3	14.29
Occupation		
Unemployed	8	38.10
Trading	3	14.29
Teaching	4	19.05
Student	6	28.57
Role in community		
Community members	12	57.14
Students	6	28.57
Executives of GFD/GBU	3	14.28

^a^Mean = 42.23 (SD = 17.15), Median = 50

#### Themes

The themes identified in the study is presented in Table 2.

**Table 2:** Key emerging themes

Themes	Sub-themes	Quotes
Theme 1: Knowledge of SRH services and care	Have heard of SRH services and care	“I have heard about condom… they say that when you are about to have a sexual encounter with a man, then he will wear it around his penis” (IDI, Participant 6)
	Sexual intercourse (between a male and a female)	“it’s the sexual relationship between males and females and the way you will protect yourselves to prevent you from being infected with diseases [Sexually Transmitted Diseases]” (FGD1, Participant 5)
	Measures used to prevent unplanned pregnancy	“like someone going to do family planning… its excessive childbearing, maybe She has giving birth to an unplanned child and She doesn’t want to give birth again so She can go and do family planning so that the pregnancy will no longer come again or someone may want to space the children, maybe their ages are closer… so she wants to space it for the children to grow” (IDI, Participant 1)
		“It’s the women who have to use it [SRHR services and care] because the man has no medicine [SRHR services and care methods] to use, the only methods he may use is condom! So it’s the woman who has to use medicine [SRH methods] (FGD1, Participant 2)
	Measures used to prevent Sexually Transmitted Diseases (STDs)	“Right now if we talk about protection, it’s not about family planning alone but also disease prevention[infectious] so it’s necessary that both the man and the woman prevent themselves… if the woman is interested in family planning then it’s the woman who should use such services… thus going to the hospital for injection or pills that you would take… and also concerning the man, there are some of them who engage in sexual intercourse with any woman they come across, so the man is also supposed to protect himself by using something like a condom” (FGD1, Participant 5)
Theme 2: Relevance of accessing SRH services and care information	Education on STDs	“What they say is that if you want to marry, it is better than you and your partner visit the hospital for blood test… when the two [man and woman] conduct the blood test and there is no disease then you can proceed to marry” (FGD1, Participant 2)
		“before a man proposed to you, the two of you need to have a common understanding to go for medical check-ups at the hospital to confirm that there is no disease before marriage” (FGD1, Participant 6)
	Education on personal hygiene	
	Education on family planning	[…] What I understand about family planning is that it protect you from unplanned pregnancy so that when you have any sexual encounter with a man you will not become pregnant (IDI Participant 5, Visually Impaired Woman)
Theme 3: Sources of accessing SRH services and care information	Formal sources (radio stations, hospitals, information centres, schools, NGOs, churches and disability association)	“I hear it from the radio and in our place, they come to the church… I asked that in case we get a cut from the needle, wouldn’t that infect us with the diseases, so the whole congregation said that you cannot be infected through that means (FGD1, Participant 2)
		“I went to the hospital and they asked me whether I have been pregnant before so he [health professional] tested me to confirm that I am not pregnant but was sick. they ask whether I have used condom before so they advise that when you use it, you will not become pregnant or protect you from unwanted pregnancy” (FGD _ Participant 2)
	Informal sources (relatives, mothers, siblings and cousins and friends	“It is my mother who educated me that I should be very careful with our sexual relationship so that we would not be infected with any disease” (FGD1 _ Participant 2)
		“They [my siblings] mentioned that when you have many children, you go for injection or insert materials in your body so that you could not give birth… some too they go for injection for every three months (IDI, Participant 1, Visually Impaired woman)
Theme 4: Content of SRH information and education	Avoidance of sharing blades and injections with others	“I went to the hospital and one nurse told us that we should take good care of our children… we shouldn’t allow the children to use blade from the ground and also we shouldn’t seek over-the-counter injections… when you take an over-the-counter injection, you can be infected with the AIDs… so we should educate our children on such issues (FGD1, Participant 2)
	Practicing safe sexual intercourse	“the doctor was educating us that we should be cautious about our sexual encounters… that we will not have sex with anyone in order not to be infected with such diseases… We shouldn’t pick blade lying on the ground” (FGD1, Participant 2)
	Avoiding unsafe abortion	
Theme 5: Barrier to accessing SRH services and care information	Lack of caregivers and support	“it may happen that you have been invited by a health worker in a durbar to talk in a community but you the blind person will not be having a caregiver to take you to that gathering to listen to what is going on” (FGD1, Participant 5)
		“it may happen that they have grouped discussing the issue and you may want to go and listen to it but you may not have a caregiver who will accompany you to the meeting grounds… that becomes a problem so that one is also a barrier” (FGD1, Participant 6)
		“our barrier is the caregivers… there are some people when you call them to accompany you then he/she will be distancing himself… so that is the biggest difficulty we encounter” (FGD1, Participant 2)

##### Theme 1: Knowledge on SRH services and care

Participants described SRH services and care as sexual intercourse between a male and a female, prevention against Sexually Transmitted Diseases (STDs) and family planning. Most participants understood that SRH services and care are used by both males and females. The females use SRH services and care to prevent unplanned pregnancy whilst males use it to prevent themselves against Sexually Transmitted Diseases. Other participants stated that SRH services and care are used only by females because there are no contraceptive methods for men except condom (see Table 2). A participant described it as follows:

*“it’s the sexual relationship between males and females and the way you will protect yourselves to prevent you from being infected with diseases [Sexually Transmitted Diseases]”* (FGD1, Participant 5).

Most participants have heard about the various forms of SRH services and care which include family planning methods and STDs (HIV prevention). Some participants explained that a condom is used to prevent pregnancy and STDs. However, none of the participants had ever used a condom or any family planning methods before (see Table 2).

##### Theme 2: Relevance of accessing SRH services and care

Most participants noted that SRH services and care are important to prevent sexually transmitted diseases, improve personal hygiene among teenagers, and prevent unplanned pregnancy. Couples are expected to conduct a blood test to ensure there are no diseases in their blood prior to marriage. The medical examination helps to prevent any future complications on the couples and their children (see Table 2). A participant echoed on this as follows:

*“What they say is that if you want to marry, it is better that you and your partner visit the hospital for blood test… when the two [man and woman] conduct the blood test and there is no disease then you can proceed to marry”* (FGD1, Participant 2).

##### Theme 3: Source of accessing information on SRH services and care

Most participants received SRH service and care information from formal sources such as radio stations, hospitals, information centres, schools, NGOs, churches and disability association (Ghana Blind Union) (Table 2). Other participants received information on SRH services and care information from informal sources, which include relatives (mothers, siblings and cousins) and friends. A participant narrated as follows:

*“It is my mother who educated me that I should be very careful with our sexual relationship so that we would not be infected with any disease”* (FGD1, Participant 2).

##### Theme 4: Content of SRH information and education

Most participants received education on SRH service and care, particularly, on how to prevent STDs (eg. HIV). The education provided focused on avoidance of sharing blades and injections with others, the use of condoms in sexual intercourse and unsafe abortion (Table 2). A participant’s narration is captured below:

*“I went to the hospital and one nurse told us that we should take good care of our children… we shouldn’t allow the children to use blade from the ground and also we shouldn’t seek over-the-counter injections… when you take an over-the-counter injection, you can be infected with the AIDs… so we should educate our children on such issues* (FGD1, Participant 2).

##### Theme 5: Barriers to accessing information on SRH services and care

Most participants faced barriers in accessing SRH service and care information. The barriers are the lack of family caregivers and support services to accommodate participants during SRH services care meetings and durbars. In some instances, family members are unwilling to accompany participants to SRH service and care centres (Table 2). A participant echoed on this as follows:

*“it may happen that you have been invited by a health worker in a durbar to talk in a community but you the blind person will not be having a caregiver to take you to that gathering to listen to what is going on”* (FGD1, Participant 5).

### Discussion

The study was conducted to explore the knowledge and sources of information about SRH services and care among visually impaired women in Ghana. The study identified five themes (see Table 2). The themes are discussed and guided by the Longo’s et al. expanded model of health information seeking behaviours, with components of contextual factors, active information seeking and passive receipt of information.

### Active information seeking

The knowledge of SRH services information is significant to influence the health-seeking behaviour of visually impaired women [1]. Active information seeking involves the awareness of and access to SRH information among visually impaired women [1, 3]. Generally, the findings from this study confirmed that visually impaired women were knowledgeable about SRH services information. The knowledge on SRH services information were limited to sexuality, family planning and STDs. The knowledge of visually impaired women on SRH services information can help to prevent STDs, unplanned pregnancies (family planning) and enhance adolescent sexual behaviour. The finding is consistent with an earlier study in LMICs [34], where people with disability recognized the benefits of accessing SRH information. The visually impaired women have some misconceptions regarding the group of individuals that need the services as well as the type of services required [8, 34, 35]. The findings imply that visually impaired women may have knowledge about SRH information but does not translate the knowledge into service use and health decision making. The findings recommend that the current advocacy and awareness of SRH issues should attempt to provide comprehensive information.

### Passive receipt of information

The sources of information about SRH service and care is relevant to inform visually impaired women about the existing services. The current findings suggest that visually impaired women access information on SRH services and care from both formal and informal sources. The formal sources are radio stations, hospitals, information centres, schools, NGOs, churches and disability association. Others received information on SRH services and care from informal sources, such as relatives and friends. This finding confirms earlier studies in other settings, including Tanzania [36], Senegal [34], Cambodia [37] and Uganda [5]. In most of these settings, visually impaired women access information on SRH services and care from both formal and informal but information from formal sources were more reliable and preferred [34, 36, 37]. Although the visually impaired women access SRH information from formal and informal sources, it is uncertain whether or not they use information from these sources to make personal health care decision. The findings recommend that future study should attempt to use an interventional study to explore the effectiveness of using formal and informal SRH information sources to improve personal health decision and well-being for visually impaired women. Further, the findings recommend that policies and strategies that aim at advocating and creating awareness about SRH services for visually impaired women should target both formal and informal sources.

### Contextual factors

The contextual factors, i.e. the strengths and weaknesses of the health system, community resources and family support services have the ability to improve access to SRH information for vulnerable population [1, 3]. However, the findings suggest that some contextual factors (family and caregiver support services) created barrier for visually impaired women when accessing SRH information. In particular, the visually impaired women lacked family and caregiver support to accompany them to service centres. Compared to high-income countries, the health systems in many LMICs including Ghana, have limited accessibility to information as well as inclusive SRH services and care for women with disability [7]. In most instances, the family caregivers appear as the only sources that can mitigate the barriers when accessing information on SRH services care. However, the lack of family caregivers support confirms that visually impaired women are likely to be frustrated at SRH services and care centres particularly due to the specific needs and nature of their disabilities. More importantly, visually impaired women require accessible information in braille format to facilitate their understanding of SRH services and care. The findings corroborate previous studies in many LMICs, including Uganda [9], Tanzania [36], Senegal [34] and Cambodia [37]. In these settings, people with disabilities, including those with visual impairment faced several challenges when accessing information on SRH services and care. These challenges include lack of confidentiality, anonymity, financial barrier, distance and provider attitudes [9]. The finding recommends that health policies in LMICs, including Ghana, should adequately address barriers faced by visually impaired women when accessing SRH services and care. Specifically, family caregivers and SRH health centres should provide adequate support services for visually impaired women regarding information on SRH service.

## Limitations

The study has limitations regarding the participants, scope and data collection instruments. The study was limited to only visually impaired women, without the perspectives of families and caregivers, health workers and health systems planners. The experiences of visually impaired women could possibly influence their responses to the interviews and affect the validity of the strength of the conclusion. Similarly, the study purposively selected 21 visually impaired women across two regions of Ghana. The interview guide was a self-developed instrument based on previous literature and theories. That notwithstanding, the study employed several scientific scrutinise such as developing of interview guide with previous literature, pre-testing of tools, informed consent as well as methodological and interpretive rigour of analysing qualitative data (credibility, transferability, dependability and confirmability) [28, 29, 31].

## Data Availability

All data supporting these findings are either contained in the manuscript or available upon request. There are no restrictions to anonymized data sources. All data collection tools, including interview guides and information and consent forms are also available upon request.

## Abbreviations

LMICs: low and middle-income countries
SRH: sexual and reproductive health
IDIs: in-depth interviews
FGD: focus group discussion
GBU: ghana Blind Union
GFD: ghana Federation of Disability Organizations
STDs: sexually Transmitted Diseases
